# Higher Frailty Levels Are Associated With Increased Improvement in Health-Related Quality of Life Following a 12-Week Medical Student-Led Walking Program

**DOI:** 10.1177/30495334251395363

**Published:** 2025-11-12

**Authors:** Madeline E. Shivgulam, Taylor Wilson, Minji Choi, Emily E. MacDonald, Olga Theou, Myles W. O’Brien

**Affiliations:** 1 Nova Scotia Health & Dalhousie University, Halifax, Canada; 2 Centre de Formation Médicale du Nouveau-Brunswick, Moncton, New Brunswick, Canada; 3 Dalhousie University, Saint John, NB, Canada; 4 Dalhousie University, Halifax, NS, Canada; 5 Université de Sherbrooke, Moncton, NB, Canada

**Keywords:** community exercise programs, geriatrics, older adults, physical activity, Walk with a Doc

## Abstract

Higher frailty, or the accumulation of health deficits, is associated with worse health-related quality of life (HRQoL). Sustaining HRQoL is vital for overall well-being. Walk with a Future Doc (WWAFD) is a student-led community-based exercise and education program that is feasible and well-received by older adults. However, it is unknown how WWAFD influences HRQoL. This study tested the hypothesis that a 12-week WWAFD education and low-impact walking program would improve HRQoL, particularly among those with higher baseline frailty levels. Community-dwelling adults participated in a 12-week, medical student-led program including a 10-min educational talk and 50 min of self-paced walking once per week. All participants completed a 65-item validated Frailty Index and the EuroQol five-dimensions questionnaire at baseline and 12-weeks. Sixty-six participants were included (age: 65 ± 7 years [range: 47–85 years]; 47 females). Although there was no change in HRQoL from baseline (0.877 ± 0.137 [range: 0.038–0.949] to follow-up 0.878 ± 0.077 [range: 0.450–0.949]; *p* = .107), frailty at baseline (0.090 ± 0.073 [range: 0.000–0.394]) was associated with both baseline HRQoL (β = −1.353, 95% CI [−1.704, −1.003], *p* < .001) and larger improvements in HRQoL (0.001 ± 0.081 [range: −0.120, 0.415]; β = .663, 95% CI [0.387, 0.880], *p* < .001). Those with a higher frailty level at baseline exhibited the greatest improvements in HRQoL following the WWAFD community exercise program.

## Introduction

As people age, they are more susceptible to the development of frailty, or the state of increased vulnerability to adverse outcomes following minor illness incidence (D. H. Kim & Rockwood, 2024). Among an aging population frailty has multifaceted implications including frequent use of healthcare services, poor quality of life, worse social vulnerability, higher rates of disability, and earlier mortality (D. H. Kim & Rockwood, 2024). Among aging adults, those with improved or maintained frailty status exhibit higher health-related quality of life (HRQoL) compared to those with worsened frailty (Oishi et al., 2025). HRQoL provides a holistic view of an individual’s well-being, considering physical, mental, and social aspects. The negative association between frailty and HRQoL (H. S. Kim et al., 2024) reflects the multifaceted impact of frailty on both physical functioning and social well-being, underscoring the potential for interventions such as exercise and social engagement to manage HRQoL (Oishi et al., 2025).

Walk with a Doc is a not-for-profit international organization that aims to increase community levels of health education, physical activity, social connectedness, and time spent outdoors (Wilson et al., 2024). Subsets of the program exist across the globe, one of which is the Walk with a Future Doc (WWAFD) program which includes weekly sessions of health-related education and exercise. WWAFD is unique in that it is led by medical students, thereby encouraging early collaboration and connection amongst healthcare trainees with future patients. Based on self-reported evidence from this intervention, WWAFD may facilitate new friendships, improve social wellness, and deliver educational components perceived as interesting and relevant (Wilson et al., 2024). Given those with higher frailty may benefit the most from leading a physically active lifestyle (Kehler et al., 2020) and the novelty of WWAFD as a sustainable, integrated community-based program for older adults, it is necessary to evaluate how this multi-component model impacts HRQoL in frail adults.

Based on the inverse association between frailty and HRQoL (H. S. Kim et al., 2024) and evidence that those with higher frailty benefit most from physical activity (Kehler et al., 2020), WWAFD may improve HRQoL broadly, with the greatest gains among individuals with higher multidimensional frailty. As such, this study tested the hypothesis that (1) higher frailty (via the deficit model) would be associated with poorer HRQoL among adults in the community, (2) that the WWAFD program will increase HRQoL, and (3) that improvements in HRQoL would be more pronounced among frailer adults.

## Methods

*Participants.* Community members from New Brunswick, Canada were recruited to attend weekly walks at a YMCA facility free of charge between 2022 and 2024. Local primary care providers interested in supporting the program were provided with recruitment posters and prescription pads to encourage their patients to attend. First and second year medical students (i.e., pre-clerkship) facilitated the program. Students met with overseeing PhD level researchers and Canadian Society for Exercise physiology Clinical Exercise Physiologist before the program and monthly thereafter to go over program procedures. Each meeting was ~1-hr in duration and covered content including session structure, facilitating participant engagement, and problem-solving any issues that arose. Program participants were included in research if they were >45 years of age and attended > 50% of sessions. Twenty-six participants were excluded: 1 ≤ 45 years of age, 22 due to missing follow-up HRQoL data, and 3 due to attendance below 50%. Participants were not excluded based on mobility or health conditions. Based on a moderate effect size (*f*^2^ = 0.2) and three predictor variables (i.e., age, sex, and baseline frailty), a sample size calculation estimated that 59 participants were needed using a multiple regression model assuming a two-tailed, α = .05 and β = 80% power (G*Power, v3.1). Verbal and written consent were acquired from all participants. All protocols and procedures conformed to the Declaration of Helsinki, except depositing data in a repository, and were approved by the Human Health Research Protection Program and the Research Ethics Board at Horizon Health Network (#4403).

### Walk With a Future Doc Program

The WWAFD is a 12-week community-based walking program, as described in detail elsewhere (Wilson et al., 2024). The program included one 60-min session 1x/week. This included a 10-min health education talk given by a medical student, followed by a 50-min self-paced walk on an indoor track. The health education talks were given on a variety of topics including diet and nutrition, sleep, hygiene, chronic pain management, stretching, balance, staying motivated to exercise, setting SMART goals, hydration, the impacts of social interaction on mental health, etc. Local primary care providers acted as program advisors by attending the sessions to walk with participants and answer questions. In addition to allowing participants to engage with healthcare providers and medical students, the walk also provided a space for participants to socialize with other community members. All participant demographics (i.e., age, sex, gender, ethnicity) were self-reported. Attendance to each of the 12 sessions and the number of laps completed at each walk were self-reported by participants. For each walk, distance covered was determined by multiplying reported laps by the 200 m track length. Attendance was verified by researcher logs, whereas laps completed were entirely self-reported. There was no missingness in data for attendance or laps. HRQoL and frailty were assessed at baseline and follow-up (12-weeks).

### HRQoL

HRQoL was assessed using the EuroQol five-dimensions five-level (EQ-5D-5L) which evaluates health quality across five domains including mobility, self-care, usual activities, pain and discomfort, and anxiety and depression (Xie et al., 2016). Each domain is scored on a scale from 1 to 5 whereby 1 indicates having no problems with that domain and 5 indicates the most severe disability. Based on this scoring, an EQ-5D summary index was derived by applying a formula that attaches values (weights) to each of the levels in each dimension. The index is calculated by deducting the appropriate weights from 1, the value for full health (i.e., state 11111). The current data was analyzed using the value set of the EQ-5D-5L for Canada (Xie et al., 2016) to derive a value between 1 (best health) and 0 (a state as bad as being dead).

### Frailty Index

The frailty index implemented in the present analysis was based off the deficit accumulation model and initially developed and validated using the CLSA dataset based on standard procedures (Blodgett et al., 2022). The 65 questions are compiled from five domains within the questionnaire: activities of daily living, mobility, self-rated health, previous falls and effort, and chronic health conditions. The individual items included were coded as 0 (no deficit) or 1 (deficit). Interval or ordinal variables were coded as a proportion of complete deficit (e.g., self-rated health has 5 options: excellent = 0, very good = 0.25, good = 0.5, fair = 0.75, poor = 1). The frailty index was calculated as the deficit score ÷ the number of deficits measured for each participant (e.g., 6.5/65 = 0.10), with a value closer to 1.00 indicating a higher degree of frailty. Two participants had some missing data from their frailty index (*n* = 1 deficit missing each) and frailty was determined from 64 items in these participants.

### Statistical Analysis

All variables were assessed for normality using a Shapiro Wilk test and confirmed non-normal (all, *p* < .001). A Wilcoxon signed-rank test was used to evaluate whether there was a change in HRQoL in the sample from baseline to follow-up. Multiple regression was used to evaluate whether frailty was associated with baseline HRQoL and ΔHRQoL (post-pre) while controlling for age and sex. Assumptions for conducting regression analyses were met. To examine which of the independent variables were most strongly related to either baseline HRQoL or changes in HRQoL, we conducted relative importance analysis in conjunction with regression analysis (Johnson & Lebreton, 2004). This allowed the estimation of the raw weight that each variable contributes to the overall model. The statistical significance of the weights was determined via 10,000 replication bootstrapping, with statistical significance denoted by 95% confidence intervals not encompassing zero. All statistical analyses were completed in Version 28 IBM SPSS Statistics (IBM Corp., Armonk, NY, USA) with a statistical significance threshold of α = .05. Data were presented as mean ± standard deviation.

## Results

Sixty-six of 92 potential participants (65 ± 7 [range: 47–85] years, *n* = 47 females, *n* = 44 males, *n* = 47 women, *n* = 44 men; Supplemental Table 1) were included in the present study. The average number of weekly sessions attended was 9.2 ± 2.1/12 (range: 6/12–12/12). The average self-reported walking distance covered by each participant was 2.7 ± 1.0 km/session. At baseline, participants’ frailty levels were 0.090 ± 0.073 (range: 0.000–0.394).

**Figure 1.**
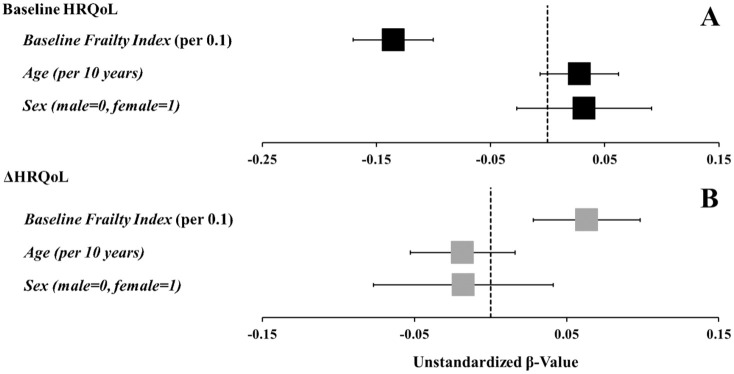
Forrest plot demonstrating the unstandardized β values and 95% confidence interval for an increase in HRQoL. Baseline frailty was associated with both baseline health-related quality of life (HRQoL) (A) as assessed by the EuroQol five-dimensions questionnaire, scored from 0 to 1, and ΔHRQoL (B) while accounting for age and sex.

In a multiple linear regression controlling for age and sex, the model explained 52.3% of the variance (*R*^2^ = .523), with pre-intervention frailty levels as a significant negative predictor of baseline HRQoL (β = −1.353, 95% CI [−1.704, −1.003], *p* < .001). Relative weights analyses confirmed that baseline frailty was the primary predictor for baseline HRQoL (frailty: 97%), while age (3%) and sex (1%) were not significant predictors as per the multiple regression (both, *p* > .111; Figure 1).

There was no statistically significant change in mean HRQoL from baseline (0.877 ± 0.137 [range: 0.038–0.949]) to follow-up (0.878 ± 0.077 [range:0.450–0.949]; *p* *=* .107; Supplemental Figure 1), with individual changes ranging from −0.120 to 0.415. Most participants (58%) showed no change in HRQoL, 27% improved, and 14% worsened. A multiple linear regression controlling for age and sex accounted for 32.9% of the variance (*R*^2^ = .329), with baseline frailty as a significant predictor for ΔHRQoL (β = .633, 95% CI [0.387, 0.880], *p* < .001, raw weight: 0.304; Figure 1B). Relative weights analysis confirmed that baseline frailty was the primary predictor for changes in HRQoL (frailty: 94%), while age (5%) and sex (1%) were not significant predictors (both, *p* > .136).

## Discussion

This study examined the ability of the WWAFD program to improve HRQoL and investigated whether frailty levels at baseline would be associated with improvements in HRQoL. Although there was no change in HRQoL between baseline and follow-up, lower baseline frailty levels were associated with higher HRQoL. Pre-intervention frailty was also associated with larger, positive changes in HRQoL because of the WWAFD program, with this effect being greater than participants’ age and sex. This emphasizes the importance of the WWAFD program, particularly amongst older adults with higher frailty levels on promoting HRQoL. These novel findings highlight the potential for multi-component community-based exercise programs to improve HRQoL amongst those who have accumulated health deficits.

Interventions designed to improve HRQoL among older adults are increasingly essential for fostering longevity and increased quality of life. These results of this study support previous evidence of an inverse association between frailty and HRQoL (H. S. Kim et al., 2024). While a previously conducted 9-month walking intervention improved HRQoL among older adult females, but not males, in the primary care setting (Villalobos et al., 2019), the current study did not observe changes in HRQoL from baseline to follow-up. Interestingly, females have higher frailty scores compared to males (Gordon et al., 2017), which may explain why it was previously observed that only females in the clinical setting improved HRQoL. These findings are consistent with previous exercise programs reporting the largest gains among participants with higher frailty levels (Kehler et al., 2020). Similarly, this study suggests that those with higher frailty may have a greater capacity to improve their HRQoL through walking-based interventions. In the current study, frailty was associated with both baseline and changes in HRQoL, but chronological age was not. This suggests that while chronological age is not a modifiable risk factor, targeting reductions in frailty may be a critical element of promoting HRQoL among the aging population. Since HRQoL represents a metric beyond healthy physiological aging and disease management, enhancing the well-being, happiness, and life satisfaction of older adults to ensure that aging is primarily about living well for longer is increasingly important. Considering the sample from the current study generally exhibited low frailty levels (i.e., 94% < 0.20) and a higher average quality of life, targeting this intervention to frailer populations, such as those living in long-term care or hospitalized adults, may increase the interventions effectiveness to improve HRQoL.

This study is strengthened by a sufficient sample size through a 12-week multi-component intervention. The feasibility of implementation of a WWAFD program, the free cost for its attendees, and its integration into the community setting, where it can elicit positive impacts on attendees, are also strengths of this medical student-led program. While the study was grounded in a community program in pragmatic conditions, the intervention is lacking a randomized controlled trial design that may increase our confidence in establishing causality. As a community-based program, this heterogeneity can pose challenges in evaluating the program’s effectiveness, as differing levels of participation (e.g., exercise dosage, attendance, etc.) may influence outcomes in complex ways. Although the sample was strengthened by variable frailty levels (range: 0.000–0.394), the current sample is also a highly homogenous convenience sample whereby it primarily consisted of Caucasian individuals living in a rural community. Future investigations are warranted to understand how a similar program may be applicable to a more diverse sample not reached.

In conclusion, the frailty levels of participants were associated with both baseline HRQoL and the change in HRQoL through a medical student-led education and exercise program after controlling for age and sex. The WWAFD community program may favorably influence HRQoL among individuals with higher levels of frailty.

## Supplemental Material

sj-docx-1-ggm-10.1177_30495334251395363 – Supplemental material for Higher Frailty Levels Are Associated With Increased Improvement in Health-Related Quality of Life Following a 12-Week Medical Student-Led Walking Program Supplemental material, sj-docx-1-ggm-10.1177_30495334251395363 for Higher Frailty Levels Are Associated With Increased Improvement in Health-Related Quality of Life Following a 12-Week Medical Student-Led Walking Program by Madeline E. Shivgulam, Taylor Wilson, Minji Choi, Emily E. MacDonald, Olga Theou and Myles W. O’Brien in Sage Open Aging
