# Supplementary material for: Higher Frailty Levels Are Associated With Increased Improvement in Health-Related Quality of Life Following a 12-Week Medical Student-Led Walking Program
Source: Sage Open Aging. 2025 Nov 12;11:30495334251395363. doi: 10.1177/30495334251395363 (PMC12612545; doi:10.1177/30495334251395363)
Supplement: sj-docx-1-ggm-10.1177_30495334251395363 – Supplemental material for Higher Frailty Levels Are Associated With Increased Improvement in Health-Related Quality of Life Following a 12-Week Medical Student-Led Walking Program [file sj-docx-1-ggm-10.1177_30495334251395363.docx]

**Supplemental Figure 1.** Health-related quality of life (assessed via the EQ-5D-5L) at baseline and 12-weeks on the WWAFD walking program in n=66 community dwelling older adults. No significant change was observed based on a Wilcoxon Signed Rank test (*p*=0.107). Individual data is plotted whereby males are depicted in solid lines and females are depicted in dashed lines.

**
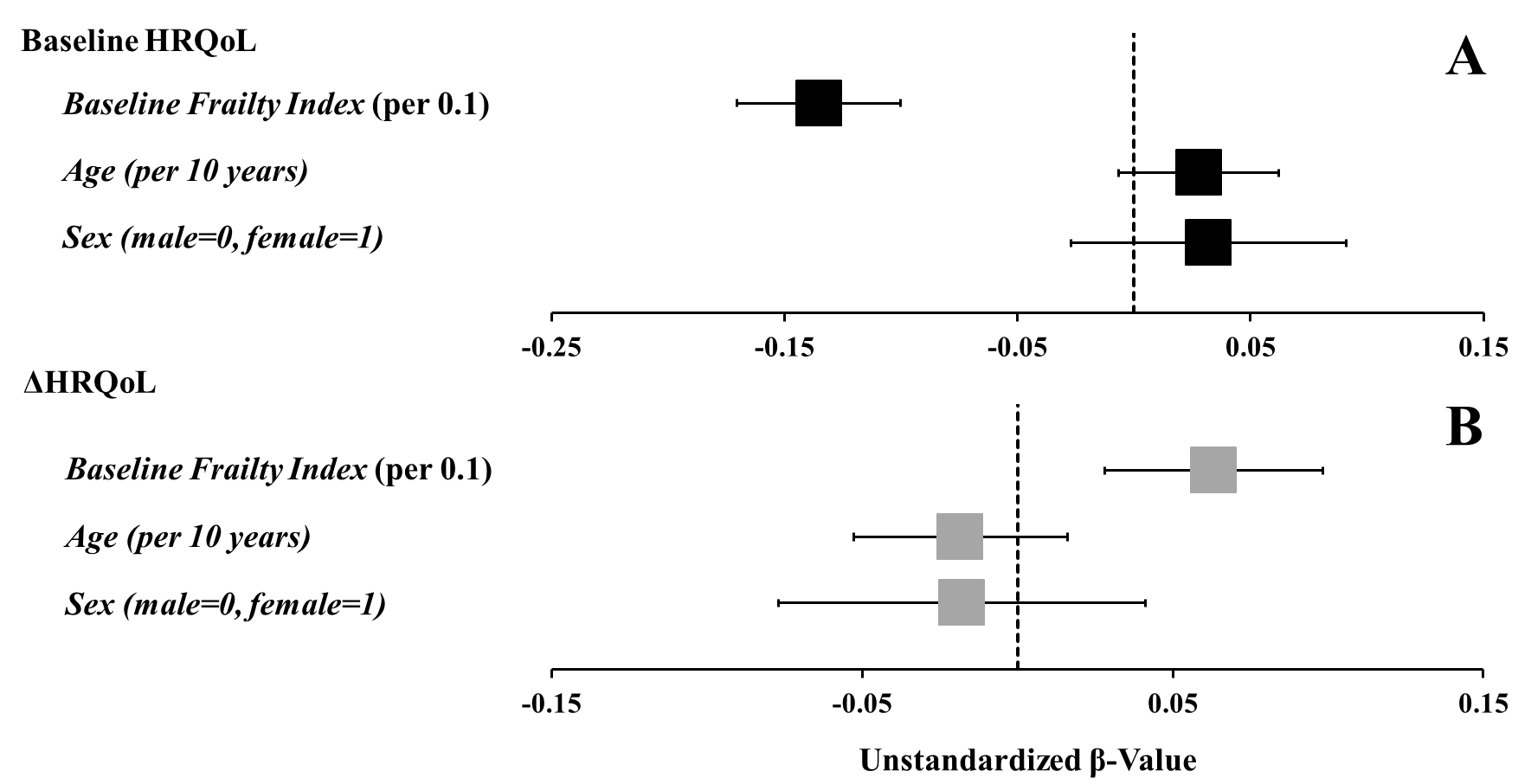
**

**-FIGURE 1-**


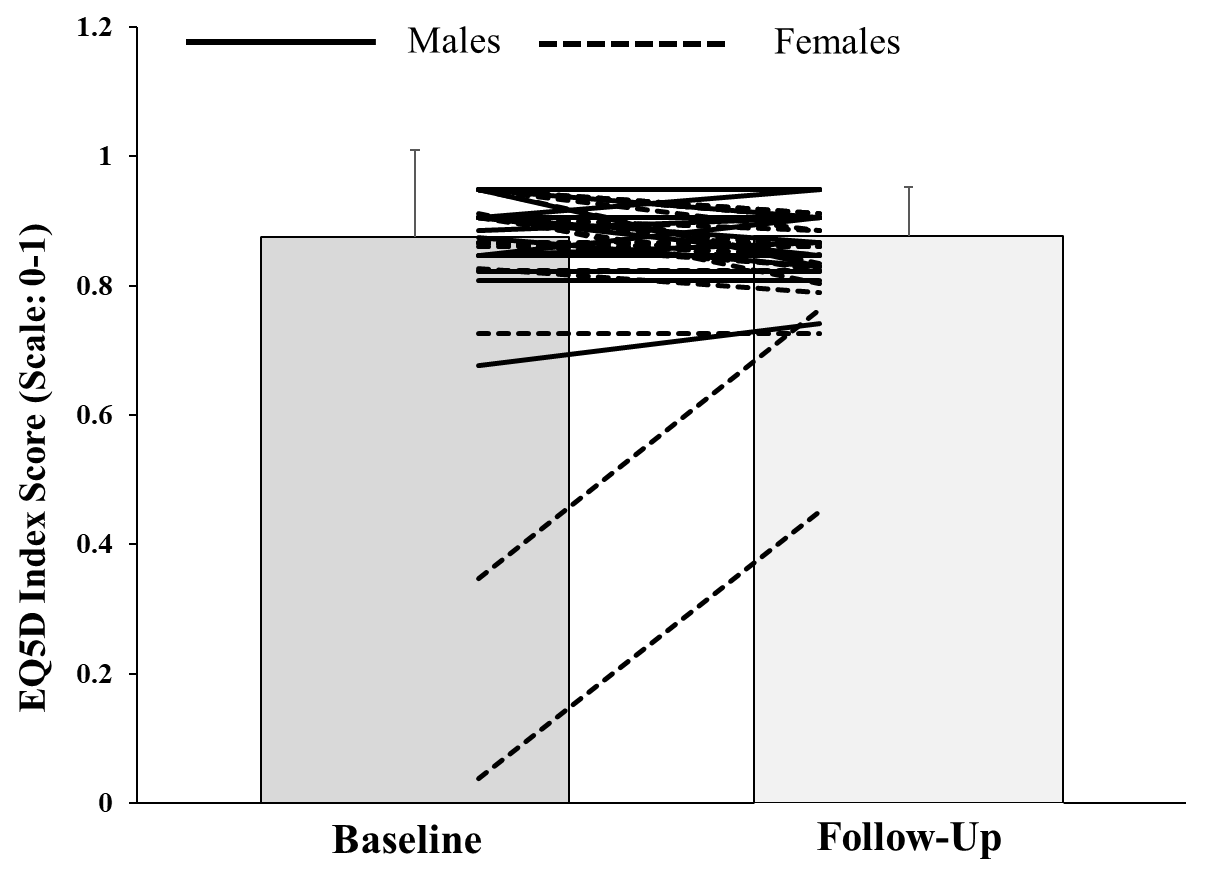


**-SUPPLEMENTAL FIGURE 1-**

| **Supplemental Table 1.** Descriptive characteristics of included participants (n=66). | |
| --- | --- |
| **Variable** | **mean±standard deviation (range)** |
| Age (years) | **65±7 (47-85)** |
| Sex (# females, males) | 47, 19 |
| Gender (women, men) | 47, 19 |
| Race (# Caucasian, African Canadian) | 65, 1 |
